# Bioactivities of Traditional Medicinal Plants in Alexandria

**DOI:** 10.1155/2018/1463579

**Published:** 2018-01-31

**Authors:** Hosam O. Elansary, Agnieszka Szopa, Paweł Kubica, Halina Ekiert, Hayssam M. Ali, Mohamed S. Elshikh, Eslam M. Abdel-Salam, Mohamed El-Esawi, Diaa O. El-Ansary

**Affiliations:** ^1^Floriculture, Ornamental Horticulture and Garden Design Department, Faculty of Agriculture (El-Shatby), Alexandria University, Alexandria, Egypt; ^2^Department of Geography, Environmental Management and Energy Studies, University of Johannesburg, APK Campus, 2006, South Africa; ^3^Department of Pharmaceutical Botany, Medical College, Jagiellonian University, Ul. Medyczna 9, 30-688 Kraków, Poland; ^4^Botany and Microbiology Department, College of Science, King Saud University, P.O. Box 2455, Riyadh 11451, Saudi Arabia; ^5^Timber Trees Research Department, Sabahia Horticulture Research Station, Horticulture Research Institute, Agriculture Research Center, Alexandria, Egypt; ^6^Botany Department, Faculty of Science, Tanta University, Tanta, Egypt; ^7^Precision Agriculture Laboratory, Department of Pomology, Faculty of Agriculture (El-Shatby), Alexandria University, Alexandria, Egypt

## Abstract

In traditional folklore, medicinal herbs play a vital role in the prevention and treatment of microbial diseases. In the present study, the phenolic profiles of the medicinal plants* Asparagus aethiopicus* L.,* Citrullus colocynthis* L.,* Senna alexandrina* L.,* Kalanchoe delagoensis* L.,* Gasteria pillansii* L.,* Cymbopogon citratus*,* Brassica juncea*, and* Curcuma longa* L. were determined by high-performance liquid chromatography with a diode-array detector method. The results revealed rich sources of important compounds such as robinin in the fruits and leaves of* A. aethiopicus*; caffeic acid in the tubers of* A. aethiopicus* and quercitrin in the leaves of* G. pillansii*. Further, relatively high antioxidant, antibacterial, and antifungal activities were observed in* C. colocynthis* fruit coat,* S. alexandrina* pods, and* A. aethiopicus* leaves, respectively. The relatively higher the bioactivities of plants extracts associated with the phenols in these plants, in particular, the more abundant the phenols. Therefore, it was concluded that the fruit coat of* C. colocynthis*, pods of* S. alexandrina*, and leaves of* A. aethiopicus* might be excellent sources of natural products. These plant extracts also have a wide spectrum of antimicrobial activities that could be used in the pharmaceutical industries and to control diseases.

## 1. Introduction

Medicinal plants represent an important part of disease remedy in economically weak regions of the world, such as Africa [1, 2]. The South Mediterranean region, including Egypt, is a rich source of medicinal plants that have several uses as alternative medicine across history [3, 4]. Some of these medicinal plants, such as mint [5–7] and basil [8, 9], have been thoroughly investigated. However, many medicinal plants, including* Asparagus aethiopicus* L. and* Citrullus colocynthis* L., have not been well studied, especially their phenolic profile and bioactivity.

The genus* Asparagus*, belonging to the family Liliaceae, contains about 300 species that are widely used as complementary and alternative medicines for treating dysuria, diabetes, epilepsy, night blindness, tumors, and dysentery. They are also used for enhancing appetite and increasing milk secretion in women [10, 11]. The species* A. aethiopicus* is common across Egypt, and few studies have investigated this species. A study on plants of the South Delta region indicated that the leaves of* A. aethiopicus* contain flavonoids and have moderate antioxidant activities [12]. Furthermore, other species of* Asparagus* have revealed great diversity in their chemical composition. Aberoumand [13] identified fatty acids in the wild* Asparagus officinalis* from Iran.

*Citrullus colocynthis* (L.) Schrad, commonly known as bitter apple, belongs to the family Cucurbitaceae and grows naturally in the Egyptian desert. The plant is traditionally used for treating cancer, leukemia, rheumatism, and amenorrhea and also used as an insect repellant [14, 15]. Gurudeeban et al. [16] reported several traditional uses of the Indian* C. colocynthis*, including its use as antidiabetic, antipyretic, anti-inflammatory, antibacterial, and antifungal agents. However, the information available on the Egyptian ecotype of* Citrullus colocynthis* is scarce in relation to its phenolic composition and bioactivities.

*Cymbopogon citratus* (Poaceae), commonly known as lemon grass, a native of Southeast Asia, is widely grown in Africa as a medicinal herb [17]. The plant is traditionally used in folk medicine as an anti-inflammatory agent and also used as an insect repellent. A recent investigation indicated the presence of polyphenolic flavonoids, including luteolin and apigenin, with anti-inflammatory activity in the leaves of* C*.* citratus* [18].

The genus* Gasteria*, a native of South Africa, contains 23 species. One of the species* G. pillansii* (L.) Haw, a succulent, is widely cultivated as an ornamental plant in Egypt [19]. Few studies have been conducted on the genus revealing the presence of dihydroanthracenones in* G. bicolor* [20].

*Kalanchoe delagoensis* L. (Crassulaceae), a succulent plant, native of Madagascar, is known to contain bufadienolide cardiac glycosides that are toxic [21]. However, thus far, studies have not been conducted to characterize the phenolic profile of this plant.

*Brassica juncea* (Brassicaceae) is traditionally used as a stimulant, a rubefacient, an emetic, and a diuretic, and it is consumed as food as well [22]. Few studies have indicated that* B. juncea* contain some phenols [23].

*Curcuma longa* (Zingiberaceae), commonly known as turmeric, is considered a medicinal plant and is also used as a spice. The roots (rhizomes) are used in traditional Indian and Chinese medicines to treat wounds, acne, flu, and urinary tract and liver diseases [24]. The roots contain phenols, such as diferuloylmethane (curcumin), that have antioxidant and anticancer properties [25, 26].

Another common medicinal plant in Egypt is* Senna alexandrina* L. (Fabaceae), which is distributed around the Nile River. Traditionally, the plant is used for treating stomach pain and constipation, because of its laxative effects [27, 28]. The pods (fruits) have a high percent of glycosides, including the sennosides A, B, C, and D, as well as anthraquinones [29, 30]. The phenolic compounds are the major phytochemicals that are universally present in plants (estimated to be 10,000 compounds) and are responsible for the bioactivity of the plant extracts [31, 32]. The search for natural products in plants is strongly associated with the presence of phenolic compounds, since they have antioxidant and antimicrobial activities [33–35]. The phenolic profile and bioactivities of the traditional medicinal plants in northern Egypt have not been fully explored. In the present study, we aimed to investigate the phenolic qualitative and quantitative profiles of the selected medicinal plants that are used in complementary and alternative medicines by the local people. Furthermore, the antioxidant activity of the extracts was evaluated by 2,2′-diphenylpicrylhydrazyl (DPPH) and *β*-carotene-linoleic acid assays. The antibacterial and antifungal activities of each plant were also evaluated to identify and elucidate the bioactivity of the identified main phenolic compounds.

## 2. Material and Methods

### 2.1. Plant Material

The leaves, fruits, and tubers of* A. aethiopicus* L. (*A. sprengeri* Regel) and leaves of both* G. pillansii* L. Haw and* K. delagoensis* L. were obtained from the Department of Floriculture and Ornamental Horticulture Nursery in El-Shatby, Alexandria. The fruit coat and flesh with seeds of* C. colocynthis* L., leaves of* C*.* citratus* (DC). Stapf, and pods of* S. alexandrina* Mill were obtained from commercial farms in Alexandria Governorate in northern Egypt. Further, the seeds of* B. juncea* L. and roots of* C. longa* L. were procured from a local herbalist. All the plants were identified by Dr. H. Elansary and vouchered at the Faculty of Agriculture, Alexandria, in June 2017.

### 2.2. Bioactivity Assays

To determine the bioactivity of the samples, the total dry weight was determined by drying the cleaned plant parts (0.25 mg) in an oven at 35°C to reach a constant weight and then weighed. The samples were then ground and dissolved in 3 mL methanol (99%) in dark condition, for 1 h, at room temperature. The extracts were centrifuged at 10,000 rpm (7000 ×g) for 5 min and the supernatant (~2.7 mL) was stored at −80°C. All the chemicals used were of analytical/HPLC grade bought from Sigma-Aldrich (Cairo, Egypt). The fungal and bacterial strains were procured from the Departments of Plant Pathology and Floriculture and Ornamental Horticulture, Faculty of Agriculture, Alexandria, Egypt.

### 2.3. HPLC-DAD Analyses

The plant materials that were dried by lyophilization (Labconco, USA) were powdered using a mortar. The plant samples of 0.2 g each were extracted following the procedure described by Szopa et al. [36]. The methanolic extracts were subjected to chromatographic analyses by a modified validated HPLC method [37–39]. Quantification was carried out by comparing the UV-DAD spectra and *t*_*r*_ values with that of the commercially available standards of the following groups of metabolites: free phenolic acids (3,4-dihydroxyphenylacetic, caffeic, chlorogenic,* o-*coumaric,* m-*coumaric,* p-*coumaric, ferulic, gallic, gentisic, hydrocaffeic,* p-*hydroxybenzoic, isoferulic, neochlorogenic, protocatechuic, rosmarinic, salicylic, sinapic, syringic, and vanillic acids; and precursors of phenolic acids: cinnamic and benzoic acids), flavonoids (aglycones: kaempferol, luteolin, myricetin, quercetin, and rhamnetin; glycosides: apigetrin, cynaroside, hyperoside, isoquercetin, quercitrin, robinin, rutin, trifolin, and vitexin), coumarins (bergapten, esculin, 6-hydroxy-4-methylcoumarin, imperatorin, isopimpinellin, marmesin, psoralen, umbelliferone, and xanthotoxin), catechins (epicatechin, epicatechin gallate, epigallocatechin, and epigallocatechin gallate), and phenolic compound biosynthetic precursors (phenylalanine and tyrosine) (the compounds were supplied by Sigma-Aldrich, Germany). An HPLC-DAD (Merck-Hitachi) and a Purospher® RP-18e analytical column (4 × 250 mm, 5 mL; Merck) were used for the analyses. The gradient program used included the flow rate of 1 mL/min, injection volume of 10 *μ*L, and detection wavelength of 254 nm [36, 40, 41]. The representative chromatograms have been provided as Supplementary Figures 1 and 2.

### 2.4. Antioxidant Activity

The antioxidant activities of all the samples were determined by DPPH and *β*-carotene-linoleic acid assays [9]. In the DPPH method, the absorbance was measured at 517 nm, following 30 min of treatment. In the *β*-carotene-linoleic acid assay, the absorbance was measured at 470 nm. The sample concentration required to scavenge 50% of the DPPH/*β*-carotene-linoleic acid (IC_50_ in *μ*g/mL) was determined by plotting the inhibition percent against extract concentration. Butylated hydroxytoluene (BHT) was used as a positive control. The antioxidant activity of each sample was compared with that of the BHT and blank. Experiments were repeated twice in triplicate.

### 2.5. Antibacterial Activity

The antibacterial activity of plant extracts was screened against Gram-positive and Gram-negative bacteria, including* Listeria monocytogenes* (clinical isolate),* Staphylococcus aureus* (ATCC 6538),* Bacillus cereus* (ATCC 14579),* Micrococcus flavus* (ATCC 10240).* Pseudomonas aeruginosa* (ATCC 27853), and* Escherichia coli* (ATCC 35210). The microdilution method was used to determine the minimum inhibitory concentration (MIC) and minimum bactericidal concentration (MBC) of the plant extracts [42]. The 96-well microtiter plates were used, and each well of the plate contained known concentration of plant extracts + 100 *μ*L tryptic soy broth containing bacterial inoculum (1.0 × 10^4^ CFU per well). These plates were incubated at 37°C for 24 h in a rotary shaker and the MICs and MBCs were determined after the incubation period. The lowest concentration of plant extract that exhibited no visible growth (observed under a binocular microscope) was defined as the MIC. The MBC was determined using serial subculturing of the 2 *μ*L plant extracts into microtiter plates containing 100 *μ*L of TSB in each well. The microtiter plates were incubated at 37°C for 24 h. The MBC was defined as the lowest concentration that caused no visible growth which indicated killing of 99.5% of the inoculum. The optical density was determined at 655 nm and all the experiments were performed twice in triplicate. Positive (streptomycin, 0.01–10 mg/mL) and negative (DMSO, 5%) controls were also used.

### 2.6. Antifungal Activity

Several fungi, including* Aspergillus flavus* (ATCC 9643),* A. ochraceus* (ATCC 12066),* A. niger* (ATCC 6275),* Penicillium ochrochloron* (ATCC 48663),* P. funiculosum* (ATCC 56755), and* Candida albicans* (ATCC 12066), were used to test the antifungal activity of leaf extracts using the microdilution method [42]. Known concentrations of each leaf extract (2 *μ*L) were added to microtiter plates (with a capacity of 96 wells) with each well containing broth Malt medium mixed with each fugal inoculum (spore suspension concentration of 1.0 × 10^5^). The microtiter plates were incubated at 28°C for 72 h in a rotary shaker. The minimum inhibitory concentrations (MICs) were calculated as the lowest concentration that inhibited fungal growth at the binocular microscopic level. The minimum fungicidal concentrations (MFCs) were calculated using a serial subcultivations of the leaf extracts (2 *μ*L) added to microtiter plates that contained 100 *μ*L of broth and inoculums and then incubated at 28°C for 72 h. The MFC was defined as the minimum concentration that caused no visible growth which indicated the killing of 99.5% of the original inoculum. The ketoconazole (KTZ) (1–3500 *μ*g/mL) was used as a positive control. All experiments were repeated twice in triplicate.

### 2.7. Statistical Analyses

Statistical analysis was performed using SPSS version 22.0, and the results were analyzed by least significance difference (LSD) in the ANOVA.

## 3. Results

### 3.1. Targeted Profiling of Biologically Active Metabolites

#### 3.1.1. *Asparagus aethiopicus*

The HPLC-DAD analysis of fruit, leaf, and tuber extracts of* A. aethiopicus* revealed the differences in the chemical composition between them (Table 1). In the fruit extracts, three phenolic acids, chlorogenic, gallic, and syringic acids, and four flavonoids, isoquercetin, quercetin, quercitrin, and robinin, were confirmed. The predominant phenolic acid was gallic acid (12.20 mg per 100 g DW), while robinin (kaempferol 3-*O-*robinoside-7-*O-*rhamnoside) (40.01 mg per 100 g DW) and quercitrin (quercetin 3-rhamnoside) (37.08 mg per 100 g DW) were the predominant flavonoids.

In the leaf extracts, four phenolic acids were detected, including caffeic, chlorogenic, gallic, and vanillic acids, with chlorogenic acid (31.72 mg per 100 g DW) being the most abundant. In the leaf extract relatively high amounts of robinin (1356.90 mg per 100 g DW), rutin (176.33 mg per 100 g DW), and apigenin (59.59 mg per 100 g DW) were confirmed, as well.

The phenolic profile of tuber extract was relatively poor and revealed four phenolic acids, of which three were similar to those of the leaf extracts (caffeic, chlorogenic, and gallic acids), in addition to protocatechuic acid. Relatively high amounts of caffeic acid (62.18 mg per 100 g DW) and gallic acid (20.33 mg per 100 g DW) were observed.

#### 3.1.2. *Citrullus colocynthis*

The extracts of* C. colocynthis* fruit parts (fruit coat, fruit flesh, and seeds) exhibited similarity in their phenolic profiles (Table 2). Five similar phenolic acids were quantified, including caffeic,* p-*coumaric, ferulic,* p-*hydroxybenzoic, and hydroxycaffeic acids. The hydroxycaffeic acid was the predominant compound (277.45, 117.46, and 97.25 mg per 100 g DW in the fruit coat, fruit flesh, and seed extracts, resp.). Further, the fruit coat and seed extracts contained relatively high amounts of 3,4-dihydroxyphenylacetic acid (221.46 and 47.08 mg per 100 g DW, resp.) and trace amounts of syringic acid (0.22 and 0.43 mg per 100 g DW). In addition to these, the biosynthetic precursor of phenolic compounds, phenylalanine (747.83 and 120.78 mg per 100 g DW, resp., in the fruit coat and seed extracts) was detected in considerable amount.

#### 3.1.3. *Cymbopogon citratus*,* Gasteria pillansii*, and* Kalanchoe delagoensis*

The phenolic profiles of the economically important part (leaves) of* C. citratus*,* G. pillansii* and* K. delagoensis* were determined (Table 3). The leaf extract of* C. citratus* revealed seven phenolic compounds, including one flavonoid (rutin, 155.52 mg per 100 g DW). The amounts of phenolic acids were relatively high, with chlorogenic (49.98 mg per 100 g DW) and 3,4-dihydroxyphenylacetic acid (60.21 mg per 100 g DW) being the predominant ones. The biosynthetic precursor of phenolic acids (cinnamic acid, 50.44 mg per 100 g DW) was also found in relatively high amounts.

The phenolic profile of* G. pillansii* leaf extracts contained several phenolic acids such as* p-*coumaric (14.11 mg per 100 g DW), ferulic (67.62 mg per 100 g DW), and gallic (15.04 mg per 100 g DW) acids, including their biosynthetic precursor, cinnamic acid (4.80 mg per 100 g DW). In the leaf extracts of* G. pillansii*, relatively high amounts of quercitrin (83.08 mg per 100 g DW) and quercetin (65.81 mg per 100 g DW) were confirmed, in addition to the other flavonoids, isoquercetin, and rutin.

The leaf extracts of* K. delagoensis* contained relatively high amounts of phenolic acids and flavonoids. Five phenolic acids were detected in significant amounts, including gallic acid (152.21 mg per 100 g DW) and caffeic acid (65.28 mg per 100 g DW). High amounts of the flavonoids such as trifolin (kaempferol 3-galactoside) (873.63 mg per 100 g DW), kaempferol (207.41 mg per 100 g DW), isoquercetin (120.58 mg per 100 g DW), and quercitrin (89.86 mg per 100 g DW) were detected.

#### 3.1.4. *Brassica juncea*

The seed extracts of* B. juncea* revealed the presence of phenolic acids, coumarins, and epigallocatechin (Table 4). The predominant phenolic acids included chlorogenic acid (45.03 mg per 100 g DW) and its isomer, neochlorogenic acid (18.60 mg per 100 g DW). Other phenolic acids (caffeic, ferulic, gallic, sinapic, syringic, and vanillic acids) were also quantified; however, they were in relatively low amounts (<10 mg per 100 g DW). In the seed extracts, two coumarins, esculin (294.90 mg per 100 g DW) and umbelliferone (277.27 mg per 100 g DW), were confirmed.

#### 3.1.5. *Curcuma longa*

The chromatographic analysis of the* C. longa* root extracts revealed the presence of seven phenolic aids, including* p-*coumaric, 3,4-dihydroxyphenylacetic, ferulic,* p-*hydroxybenzoic, hydroxycaffeic, protocatechuic, and syringic acids. The predominant compounds were hydroxycaffeic acid (64.40 mg per 100 g DW), 3,4-dihydroxyphenylacetic acid (33.26 mg per 100 g DW), and ferulic acid (15.04 mg per 100 g DW).

#### 3.1.6. *Senna alexandrina*

The pod extract* of S. alexandrina* contained three groups of secondary metabolites, including phenolic acids, flavonoids, and coumarins (Table 6). The precursor of the phenolic acids, benzoic acid, was quantified in relatively high amount (369.15 mg per 100 g DW). Also, seven phenolic acids (caffeic, gallic, gentisic, neochlorogenic, protocatechuic, syringic, and vanillic acids) were detected. High amounts of gentisic acid (363.21 mg per 100 g DW), neochlorogenic acid (65.15 mg per 100 g DW), and protocatechuic acid (31.52 mg 100 g DW) were detected.

Furthermore, seven flavonoids (cynaroside, isoquercetin, isorhamnetin, kaempferol, luteolin, quercetin, and rhamnetin) were detected in relatively high amounts ranging from 22.81 to 291.30 mg per 100 g DW. The predominant compounds were rhamnetin (291.30 mg per 100 g DW), isoquercetin (195.15 mg per 100 g DW), and kaempferol (137.74 mg per 100 g DW). In the* S. alexandrina* pod extracts, two coumarins, 6-hydroxy-4-methylcoumarin (62.72 mg per 100 g DW) and psoralene (20.93 mg per 100 g DW), were also detected.

### 3.2. Antioxidant Activities

Methanolic extracts of the leaves were subjected to DPPH and*β*-carotene-linoleic acid assays, and the results are presented in Table 4. The antioxidant activities expressed as IC_50_ ranged from 2.4 to 8.4 *μ*g/mL and 2.3 to 9.9 *μ*g/mL in the DPPH and *β*-carotene-linoleic acid assays, respectively.

Relatively high antioxidant activities (low IC_50_, *μ*g/mL) was exhibited by* C. colocynthis* fruit coat (DPPH = 2.4;*β*-carotene = 2.3),* S. alexandrina* pods (DPPH = 2.6;*β*-carotene = 2.4), and* A. aethiopicus* leaves (DPPH = 3.1;*β*-carotene = 2.7). They showed higher antioxidant activities than that of the BHT standard. Other plant samples of* K. delagoensis*,* G. pillansii*, and* B. juncea* revealed moderate antioxidant activities. Relatively low antioxidant activities were observed in* A. aethiopicus* fruits and tubers,* C. colocynthis* fruit flesh and seeds,* C. longa* roots, and* C. citratus* leaves. Both the assays followed a similar pattern in their values for the antioxidant activities.

### 3.3. Antibacterial Activities

The plant parts showed great variation in the antibacterial activities, expressed as MIC and MBC, of their methanolic extracts (Table 5). The MIC and MBC ranged from 0.02 to 0.43 mg/mL and 0.4 to 0.73 mg/mL, respectively. The pods of* S. alexandrina*,* C. colocynthis* fruit coat, and* A. aethiopicus* leaves revealed relatively high antibacterial activities, with relatively low MIC and MBC compared to streptomycin. The fruit flesh and seeds of* C. colocynthis* showed moderate to high antibacterial activities against bacteria, such as* P. aeruginosa*,* E. coli*,* M. flavus*, and* S. aureus*. The seeds of* B. juncea* and roots of* C. longa* revealed high to moderate antibacterial activities when compared with other plants. The tubers of* A. aethiopicus* exhibited relatively low antibacterial activity against most organisms.

### 3.4. Antifungal Activities

The antifungal activities of methanolic extracts were expressed as MIC and MFC (Table 6). The MIC and MFC values ranged from 0.08 to 0.33 mg/mL and 0.16 to 0.75 mg/mL, respectively. The fruit coat of* C. colocynthis*, pods of* S. alexandrina*, and leaves of* A. aethiopicus* showed relatively high antifungal activities in general with low MIC and MBC values compared to the standard reagent. The other plant extracts, such as* C. colocynthis* fruit flesh and seeds,* B. juncea* seeds, and* C. longa* roots, exhibited moderate antifungal activities.

## 4. Discussion

The extracts of* A. aethiopicus* (leaves, fruits, and tubers),* G. pillansii* (leaves),* S. alexandrina* (pods),* C. colocynthis* (fruit coat and flesh and seeds),* K. delagoensis* (leaves),* C. citratus* (leaves),* B. juncea* (seeds), and* C. longa* (roots) revealed significant variations in their phenol profile (Tables 1–6). The major compound found in the leaves of* A. aethiopicus* was the glucoside flavonoid, robinin (1356.90 mg per 100 g DW). Its concentration was significantly higher than that of all other compounds detected in the investigated samples. The sources of robinin, in nature, are limited; few investigations have detected this flavonoid in some plants, such as* Pueraria lobata* [43]. However, the present study revealed a rich and new source of robinin, the leaves of* A. aethiopicus*. Previous investigations on* A. aethiopicus* have isolated other flavonoids, including apigenin, apigenin-7-*O-*glucoside, dihydroquercetin, and naringenin, and they also reported moderate antioxidant activity of the alcoholic extract [12]. However, in the present study, 12 phenolic compounds were isolated from the leaves, fruits, and tubers, in addition to apigenin in relatively low amount in the leaves.

In* C. colocynthis*, the fruit coat had higher phenylalanine content when compared with other extracts, including the seed and fruit flesh of* colocynthis*. A previous study has reported the presence of phenylalanine in the seeds of the Indian* C. colocynthis* [16]. However, the present study was the first to report six times higher phenylalanine content in the fruit coat of* C. colocynthis* than that of the seeds in the Egyptian ecotype. Further, the fruit coat contained other major compounds, such as 3,4-dihydroxyphenylacetic acid and hydroxycaffeic acid. The pods of* S. alexandrina* contained high amounts of gentisic acid, benzoic acid, rhamnetin, isoquercetin, and kaempferol, and the presence of these compounds have not been reported. However, Franz [27] reported the presence of other glycosides, belonging to the anthraquinone family, in* S. alexandrina*. In the present study,* B. juncea* seeds contained high amounts of esculin, umbelliferone, epigallocatechin, and chlorogenic acid. A previous study conducted on the extracts of Indian* B. juncea* confirmed the presence of sinigrin, quercetin, vanillin, and catechin [23].

The presence of phenolic compounds, such as hydroxycaffeic acid in* C. longa* roots; rutin, 3,4-dihydroxyphenylacetic acid, chlorogenic acid, and cinnamic acid in* C. citratus*; quercitrin, ferulic acid, and quercetin in* G. pillansii*; trifoline, kaempferol, quercitrin, gallic acid, and isoquercetin in* K. delagoensis* have not been reported before this study. However, a study on* C. citratus* reported the presence of specific flavonoids with anti-inflammatory activity, such as carlinoside, isoorientin, cynaroside, and luteolin [18]. Furthermore, 3,4-dihydroxyphenylacetic acid is considered as a metabolite of curcumin and has been reported in* C. longa* [26].

High antioxidant activity is usually associated with the phenols in the plants, especially the more abundant phenols [9, 44, 45]. Phenols are the secondary metabolites of plants with obvious antioxidant activities. Flavonoids, a class of phenols, such as kaempferol, tripholine, astragalin, robinin, and quercetin, have strong antioxidants that limit free radical accumulation and scavenge free radicals [46]. In the present study, relatively high antioxidant activities were found in* C. colocynthis* fruit coat,* S. alexandrina* pods, and* A. aethiopicus*, and the major compounds detected in these plant extracts were phenylalanine, benzoic acid, and robinin, respectively. Recent studies indicated that phenylalanine and benzoic acid might be strong antioxidants [47, 48]. Further, the fruit coat of* C. colocynthis*, with relatively high 3,4-dihydroxyphenylacetic content in the present study, has been associated with strong antioxidant activity [49]. Lau et al. [43] reported high antioxidant activity of* Pueraria lobata* containing high amounts of robinin, which is analogous to the findings of the present study. The high antioxidant activity exhibited by* A. aethiopicus* might be attributed to the high robinin content. The leaves of* A. aethiopicus* contained other phenolic compounds, such as rutin and chlorogenic acid, and these compounds have been known to have strong antioxidant activities [50].

Previous investigations [51, 52] have indicated that the phenols and flavonoids present in the plants may have an influence on the microorganism growth. Generalić et al. [51] reported that leaf extracts of* Salvia officinalis* L. contained high amounts of phenols and flavonoids exhibiting high antibacterial activity, especially against* E. coli*,* S. aureus*, and* B. cereus*. In the present study, relatively high antibacterial activities were discovered in the extracts of* S. alexandrina* pods,* C. colocynthis* fruit coat, and* A. aethiopicus* leaves. The phenylalanine in the* C. colocynthis* fruit coat has been reported to have antibacterial activity [53]. Ali et al. [54] reported that the Weinreb amides 3, 4, and 5 of alanine and phenylalanine exhibited good activity against* E. coli* and* Pseudomonas aeruginosa*; however, poor results were observed against* S. aureus* and* B. subtilis*. Further, Gurudeeban et al. [16] reported high antibacterial activity of the methanolic extracts of* C. colocynthis* fruits against bacteria, such as* B. subtilis*,* Streptococcus pyogenes*, and* Salmonella typhi*. Robinin extracted from* Robinia pseudoacacia* L. has high antibacterial activity [55, 56]. Benzoic acid and its derivatives have antibacterial activities and might be used as food preservatives [57]. In the present study, the fruit coat of* C. colocynthis*, pods of* S. alexandrina*, and leaves of* A. aethiopicus* showed relatively high antifungal activities in general, which might be attributed to high amounts of specific phenols in these plants. This is in agreement with the results of previous investigations [58, 59]. Zhang et al. [60] reported high antifungal activities of* Robinia pseudoacacia* extracts, and this was attributed to high robinin content. Further, Al-Snafi [61] emphasized the antibacterial and antifungal activities of the seeds of* C. colocynthis*. However, the present study was the first to explore the profile of these important phenols in* C. colocynthis* fruit parts. Further, Gurudeeban et al. [16] reported strong antifungal activities of* C. colocynthis* against* Aspergillus fumigatus* and* Mucor* sp.

## 5. Conclusions

The plants studied revealed great diversity in their phenolic profile and also their antioxidant, antibacterial, and antifungal activities. The major phenols identified were robinin in the leaves and fruits of* A. aethiopicus*, caffeic acid in* A. aethiopicus* (tubers), quercitrin in* G. pillansii* (leaves), benzoic acid in* S. alexandrina* (pods), phenylalanine in* C. colocynthis* (fruit coat and seeds), hydroxycaffeic acid in* C. colocynthis* (fruit flesh), trifoline in* K. delagoensis* (leaves), rutin in* C. citratus* (leaves), esculin in* B. juncea* (seeds), and hydroxycaffeic acid in* C. longa* (roots). The leaves of* A. aethiopicus* had relatively high amount of the phenolic compound, robinin compared with the other plants studied. Relatively high antioxidant, antibacterial, and antifungal activities were observed in* C. colocynthis* fruit coat,* S. alexandrina* pods, and* A. aethiopicus*. The high bioactivity of plant extracts was associated with the presence of phenols, especially the more abundant phenols: phenylalanine, benzoic acid, and robinin, respectively. Further, it could be concluded that the fruit coat of* C. colocynthis*, pods of* S. alexandrina*, and parts of* A. aethiopicus* might be excellent sources of natural products that could be used in the pharmaceutical industries and control of diseases.

## Figures and Tables

**Table 1:** Chemical composition of *Asparagus aethiopicus* and *Citrullus colocynthis economic plant parts*.

Plant part	Chemical compound	Amount [mg 100 g^−1^] D.W.
*Asparagus aethiopicus *(Fruit)	Chlorogenic acid	5.21 ± 0.36
	Gallic acid	12.20 ± 0.06
	Syringic acid	2.68 ± 0.92
	Quercetin	12.61 ± 0.00
	Quercitrin	37.08 ± 0.04
	Isoquercetin	7.44 ± 0.04
	Robinin	40.01 ± 0.06

*A. aethiopicus* (tubers)	Chlorogenic acid	3.17 ± 0.76
	Gallic acid	20.33 ± 0.15
	Caffeic acid	62.18 ± 1.03
	Protocatechuic acid	8.01 ± 0.01

*A. aethiopicus* (leaves)	Chlorogenic acid	31.72 ± 4.24
	Gallic acid	10.39 ± 2.17
	Caffeic acid	11.15 ± 2.97
	Vanillic acid	5.98 ± 0.66
	Robinin	1356.90 ± 2.18
	Apigenin	59.59 ± 6.11
	Rutin	176.33 ± 0.47

*Citrullus colocynthis* (fruit coat)	Ferulic acid	2.02 ± 0.09
	3,4-Dihydroxyphenylacetic acid	221.46 ± 3.13
	p-Hydroxybenzoic acid	2.26 ± 0.16
	Hydroxycaffeic acid	277.45 ± 15.66
	Caffeic acid	4.31 ± 0.95
	p-Coumaric acid	2.80 ± 0.01
	6-Hydroxy-4-methylcoumarin	58.86 ± 0.23
	Syringic acid	0.22 ± 0.01
	Phenylalanine	747.83 ± 4.99

*C. colocynthis* (fruit flesh)	Ferulic acid	3.61 ± 0.06
	p-Hydroxybenzoic acid	5.51 ± 0.24
	Hydroxycaffeic acid	117.46 ± 17.16
	Caffeic acid	13.36 ± 2.88
	p-Coumaric acid	14.47 ± 2.39

*C. colocynthis* (Seeds)	Ferulic acid	5.76 ± 0.68
	3,4-Dihydroxyphenylacetic acid	47.08 ± 0.10
	p-Hydroxybenzoic acid	12.68 ± 0.24
	Hydroxycaffeic acid	97.25 ± 0.93
	Caffeic acid	4.18 ± 0.09
	p-Coumaric acid	2.83 ± 0.30
	Protocatechuic acid	2.25 ± 0.01
	Syringic acid	0.43 ± 0.05
	Phenylalanine	120.78 ± 1.47

**Table 2:** Chemical composition of *Senna alexandrina, Brassica juncea*, and *Curcuma longa *economic plant parts.

Plant part	Chemical compound	Amount [mg 100 g^−1^] D.W.
*Senna alexandrina (pods or fruits)*	Gallic acid	15.94 ± 0.64
	Gentisic acid	363.21 ± 18.64
	Caffeic acid	13.34 ± 0.51
	Neochlorogenic acid	65.15 ± 3.72
	Protocatechuic acid	31.52 ± 1.86
	Syringic acid	5.88 ± 0.23
	Vanillic acid	5.06 ± 0.09
	Epigallocatechin	187.50 ± 0.20
	Benzoic acid	369.15 ± 15.89
	6-Hydroxy-4-methylcoumarin	62.72 ± 3.70
	Cynaroside	73.28 ± 2.82
	Kaempferol	137.74 ± 0.52
	Quercetin	80.06 ± 3.20
	Isoquercetin	195.15 ± 7.41
	Luteolin	22.81 ± 1.34
	Rhamnetin	291.30 ± 8.73
	Psoralene	20.93 ± 0.27
	Isorhamnetin	51.31 ± 0.99

*Brassica juncea (seeds)*	Chlorogenic acid	45.03 ± 4.53
	Gallic acid	9.14 ± 0.06
	Ferulic acid	9.49 ± 0.11
	Caffeic acid	5.21 ± 0.31
	Neochlorogenic acid	18.60 ± 0.02
	Syringic acid	2.04 ± 0.22
	Vanillic acid	4.46 ± 0.08
	Esculin	294.90 ± 0.63
	Umbelliferone	277.27 ± 9.69
	Sinapic acid	5.13 ± 4.32
	Epigallocatechin	45.12 ± 0.03

*Curcuma longa L. (roots)*	Ferulic acid	15.04 ± 1.49
	3,4-Dihydroxyphenylacetic acid	33.26 ± 0.51
	p-Hydroxybenzoic acid	2.77 ± 0.39
	Hydroxycaffeic acid	64.40 ± 1.60
	p-Coumaric acid	8.11 ± 2.62
	Protocatechuic acid	2.93 ± 0.03
	Syringic acid	2.22 ± 0.01

**Table 3:** Chemical composition of *Cymbopogon citratus*, *Gasteria pillansii*, and *Kalanchoe delagoensis* economic plant parts.

Plant part	Chemical compound	Amount [mg 100 g^−1^] D.W.
*Cymbopogon citratus* (leaves)	Chlorogenic acid	49.98 ± 0.62
	Cinnamic acid	50.44 ± 0.03
	3,4-Dihydroxyphenylacetic acid	60.21 ± 0.60
	Caffeic acid	8.74 ± 0.02
	Protocatechuic acid	4.36 ± 0.02
	Vanillic acid	2.95 ± 0.03
	Rutin	155.52 ± 0.32

*Gasteria pillansii/G. maculata* (leaves)	Cinnamic acid	4.80 ± 1.23
	Ferulic acid	67.62 ± 2.25
	Gallic acid	15.04 ± 0.01
	Ferulic acid	27.15 ± 0.05
	p-Coumaric acid	14.11 ± 5.54
	Quercetin	65.81 ± 4.50
	Isoquercetin	8.04 ± 0.89
	Quercitrin	83.08 ± 11.05
	Rutin	41.83 ± 5.04

*Kalanchoe delagoensis *L. or* Bryophyllum delagoense *L. (leaves)	Ferulic acid	25.92 ± 0.12
	Gallic acid	152.21 ± 0.45
	Caffeic acid	65.28 ± 0.71
	Protocatechuic acid	32.38 ± 0.11
	Syringic acid	11.90 ± 0.62
	Trifoline	873.63 ± 2.82
	Kaempferol-7 o-rhamnoside	47.65 ± 0.42
	Kaempferol	207.41 ± 0.94
	Quercetin	89.86 ± 1.49
	Isoquercetin	120.58 ± 1.01
	Robinin	125.69 ± 1.98
	Quercitrin	155.65 ± 0.88

**Table 4:** DPPH and *β*-carotene-linoleic acid assay of *A. aethiopicus*, *C. colocynthis*, *S. alexandrina*, *B. juncea*, *C. longa*, *C. citratus*, *G. pillansii, *and* K. delagoensis *methanolic extracts. Values are expressed as mean of triplicate determinations ± sd.

	DPPH free radical scavenging activity (IC_50_, *μ*g mL^−1^)	*β*-Carotene-linoleic acid assay (IC_50_, *μ*g mL^−1^)
*Asparagus aethiopicus* (leaves)	3.1 ± 0.1	2.7 ± 0.1
*Asparagus aethiopicus* (fruit)	8.4 ± 0.1	9.9 ± 0.1
*Asparagus aethiopicus* (tubers)	8.1 ± 0.1	9.5 ± 0.1
*Citrullus colocynthis* (fruit coat)	2.4 ± 0.1	2.3 ± 0.1
*Citrullus colocynthis* (fruit flesh)	6.5 ± 0.2	7.8 ± 0.3
*Citrullus colocynthis seeds *	7.8 ± 0.1	8.9 ± 0.3
*Senna alexandrina* (pods or fruit)	2.6 ± 0.1	2.4 ± 0.1
*Brassica juncea* (seeds)	5.4 ± 0.1	6.2 ± 0.1
*Curcuma longa* L. (roots)	7.3 ± 0.3	8.2 ± 0.1
*Cymbopogon citratus* (leaves)	8.3 ± 0.3	9.4 ± 0.3
*Gasteria pillansii* (leaves)	5.4 ± 0.1	6.5 ± 0.1
*Kalanchoe delagoense* L. (leaves)	4.1 ± 0.1	4.9 ± 0.1
BHT	2.9 ± 0.2	2.6 ± 0.1

**Table 5:** Minimum inhibitory (MIC) and bactericidal concentration (MBC) of *A. aethiopicus*, *C. colocynthis*, *S. alexandrina*, *B. juncea*, *C. longa*, *C. citratus*, *G. pillansii*,and* K. delagoensis *methanolic extracts (mg mL^−1^).

	*Pseudomonas aeruginosa*	*Bacillus cereus*	*Listeria monocytogenes*	*Escherichia coli*	*Micrococcus flavus*	*Staphylococcus aureus*
	MIC	MIC	MIC	MIC	MIC	MIC
	MBC	MBC	MBC	MBC	MBC	MBC
*Asparagus aethiopicus *(leaves)	0.04 ± 0.01	0.11 ± 0.01	0.12 ± 0.01	0.08 ± 0.01	0.11 ± 0.01	0.12 ± 0.01
	0.07 ± 0.01	0.20 ± 0.03	0.26 ± 0.01	0.19 ± 0.01	0.21 ± 0.03	0.27 ± 0.03
*Asparagus aethiopicus *(fruit)	0.08 ± 0.01	0.25 ± 0.01	0.36 ± 0.01	0.36 ± 0.01	0.43 ± 0.01	0.25 ± 0.01
	0.18 ± 0.02	0.57 ± 0.03	0.63 ± 0.02	0.77 ± 0.02	0.85 ± 0.03	0.57 ± 0.03
*Asparagus aethiopicus *(tubers)	0.07 ± 0.01	0.25 ± 0.01	0.27 ± 0.01	0.18 ± 0.01	0.22 ± 0.01	0.31 ± 0.01
	0.14 ± 0.01	0.57 ± 0.03	0.56 ± 0.01	0.30 ± 0.01	0.48 ± 0.03	0.73 ± 0.03
*Citrullus colocynthis *(fruit coat)	0.03 ± 0.01	0.21 ± 0.01	0.14 ± 0.01	0.08 ± 0.01	0.10 ± 0.01	0.18 ± 0.01
	0.06 ± 0.01	0.38 ± 0.03	0.27 ± 0.03	0.17 ± 0.01	0.21 ± 0.01	0.42 ± 0.03
*Citrullus colocynthis *(fruit flesh)	0.04 ± 0.01	0.18 ± 0.01	0.23 ± 0.01	0.11 ± 0.01	0.13 ± 0.01	0.19 ± 0.01
	0.08 ± 0.03	0.41 ± 0.03	0.47 ± 0.01	0.25 ± 0.03	0.27 ± 0.03	0.41 ± 0.03
*Citrullus colocynthis Seeds*	0.05 ± 0.01	0.15 ± 0.01	0.27 ± 0.01	0.10 ± 0.01	0.15 ± 0.02	0.11 ± 0.02
	0.10 ± 0.01	0.28 ± 0.03	0.49 ± 0.01	0.21 ± 0.01	0.37 ± 0.03	0.19 ± 0.03
*Senna alexandrina* (pods or fruit)	0.02 ± 0.01	0.06 ± 0.01	0.12 ± 0.01	0.09 ± 0.01	0.11 ± 0.01	0.06 ± 0.01
	0.04 ± 0.01	0.12 ± 0.01	0.29 ± 0.03	0.18 ± 0.03	0.22 ± 0.01	0.21 ± 0.01
*Brassica juncea* (seeds)	0.04 ± 0.01	0.19 ± 0.02	0.27 ± 0.01	0.15 ± 0.01	0.12 ± 0.02	0.18 ± 0.02
	0.07 ± 0.01	0.42 ± 0.03	0.48 ± 0.01	0.37 ± 0.01	0.23 ± 0.03	0.45 ± 0.03
*Curcuma longa* L. (roots)	0.04 ± 0.01	0.07 ± 0.01	0.20 ± 0.01	0.14 ± 0.01	0.11 ± 0.02	0.19 ± 0.02
	0.08 ± 0.01	0.13 ± 0.00	0.43 ± 0.01	0.32 ± 0.01	0.21 ± 0.01	0.36 ± 0.03
*Cymbopogon citratus* (leaves)	0.09 ± 0.01	0.15 ± 0.01	0.20 ± 0.01	0.15 ± 0.01	0.13 ± 0.02	0.25 ± 0.02
	0.17 ± 0.01	0.33 ± 0.03	0.41 ± 0.01	0.41 ± 0.01	0.27 ± 0.01	0.47 ± 0.01
*Gasteria pillansii* (leaves)	0.05 ± 0.01	0.17 ± 0.01	0.25 ± 0.01	0.17 ± 0.01	0.17 ± 0.01	0.17 ± 0.01
	0.09 ± 0.01	0.30 ± 0.01	0.51 ± 0.01	0.31 ± 0.01	0.30 ± 0.01	0.30 ± 0.01
*Kalanchoe delagoense* L. (leaves)	0.09 ± 0.01	0.11 ± 0.01	0.16 ± 0.01	0.13 ± 0.01	0.11 ± 0.01	0.21 ± 0.01
	0.17 ± 0.01	0.21 ± 0.01	0.33 ± 0.01	0.28 ± 0.01	0.20 ± 0.01	0.37 ± 0.03
Streptomycin	0.07 ± 0.01	0.08 ± 0.03	0.15 ± 0.01	0.13 ± 0.01	0.12 ± 0.01	0.20 ± 0.01
	0.17 ± 0.01	0.16 ± 0.01	0.30 ± 0.03	0.29 ± 0.03	0.23 ± 0.02	0.35 ± 0.01

**Table 6:** Minimum inhibitory (MIC) and fungicidal concentration (MFC) of *A. aethiopicus*, *C. colocynthis*, *S. alexandrina*, *B. juncea*, *C. longa*, *C. citratus*, *G. pillansii*,and* K. delagoensis *methanolic extracts (mg mL^−1^).

	*Aspergillus flavus*	*Aspergillus ochraceus*	*Aspergillus niger*	*Candida albicans*	*Penicillium funiculosum*	*Penicillium ochrochloron*
	MIC	MIC	MIC	MIC	MIC	MIC
	MFC	MFC	MFC	MFC	MFC	MFC
*Asparagus aethiopicus *(leaves)	0.10 ± 0.01	0.11 ± 0.01	0.09 ± 0.01	0.15 ± 0.01	0.13 ± 0.01	0.15 ± 0.01
	0.21 ± 0.01	0.25 ± 0.03	0.18 ± 0.01	0.30 ± 0.02	0.27 ± 0.03	0.31 ± 0.03
*Asparagus aethiopicus *(fruit)	0.21 ± 0.01	0.22 ± 0.01	0.33 ± 0.01	0.25 ± 0.01	0.21 ± 0.01	0.22 ± 0.01
	0.67 ± 0.01	0.43 ± 0.03	0.75 ± 0.01	0.52 ± 0.01	0.45 ± 0.03	0.45 ± 0.03
*Asparagus aethiopicus *(tubers)	0.33 ± 0.01	0.30 ± 0.01	0.15 ± 0.01	0.33 ± 0.01	0.25 ± 0.01	0.27 ± 0.01
	0.72 ± 0.01	0.68 ± 0.03	0.27 ± 0.01	0.81 ± 0.01	0.53 ± 0.03	0.58 ± 0.03
*Citrullus colocynthis *(fruit coat)	0.08 ± 0.01	0.09 ± 0.01	0.10 ± 0.01	0.11 ± 0.01	0.15 ± 0.01	0.14 ± 0.01
	0.19 ± 0.01	0.21 ± 0.01	0.21 ± 0.01	0.25 ± 0.01	0.31 ± 0.01	0.31 ± 0.01
*Citrullus colocynthis *(fruit flesh)	0.20 ± 0.01	0.21 ± 0.01	0.13 ± 0.01	0.18 ± 0.01	0.17 ± 0.01	0.22 ± 0.01
	0.41 ± 0.01	0.43 ± 0.03	0.27 ± 0.03	0.40 ± 0.03	0.36 ± 0.03	0.45 ± 0.03
*Citrullus colocynthis Seeds*	0.23 ± 0.01	0.25 ± 0.02	0.15 ± 0.01	0.17 ± 0.01	0.18 ± 0.02	0.19 ± 0.01
	0.49 ± 0.01	0.52 ± 0.03	0.31 ± 0.01	0.38 ± 0.01	0.38 ± 0.03	0.37 ± 0.03
*Senna alexandrina* (pods or fruit)	0.09 ± 0.01	0.15 ± 0.01	0.08 ± 0.01	0.11 ± 0.01	0.21 ± 0.01	0.15 ± 0.01
	0.21 ± 0.01	0.33 ± 0.00	0.16 ± 0.03	0.23 ± 0.03	0.43 ± 0.03	0.31 ± 0.01
*Brassica juncea* (seeds)	0.19 ± 0.01	0.15 ± 0.01	0.12 ± 0.01	0.20 ± 0.01	0.15 ± 0.01	0.23 ± 0.01
	0.40 ± 0.01	0.31 ± 0.03	0.25 ± 0.01	0.43 ± 0.03	0.31 ± 0.03	0.47 ± 0.03
*Curcuma longa* L. (roots)	0.10 ± 0.01	0.13 ± 0.02	0.13 ± 0.01	0.16 ± 0.01	0.16 ± 0.01	0.28 ± 0.01
	0.19 ± 0.01	0.25 ± 0.03	0.27 ± 0.01	0.33 ± 0.01	0.33 ± 0.03	0.57 ± 0.03
*Cymbopogon citratus* (leaves)	0.20 ± 0.01	0.11 ± 0.01	0.12 ± 0.01	0.11 ± 0.01	0.19 ± 0.01	0.19 ± 0.01
	0.40 ± 0.01	0.21 ± 0.03	0.23 ± 0.01	0.22 ± 0.03	0.41 ± 0.03	0.40 ± 0.03
*Gasteria pillansii* (leaves)	0.25 ± 0.01	0.10 ± 0.02	0.14 ± 0.01	0.25 ± 0.01	0.25 ± 0.02	0.27 ± 0.01
	0.57 ± 0.01	0.20 ± 0.03	0.27 ± 0.01	0.55 ± 0.01	0.56 ± 0.03	0.63 ± 0.03
*Kalanchoe delagoense* L. (leaves)	0.21 ± 0.01	0.17 ± 0.01	0.15 ± 0.01	0.20 ± 0.01	0.21 ± 0.01	0.25 ± 0.01
	0.41 ± 0.01	0.33 ± 0.03	0.29 ± 0.01	0.43 ± 0.03	0.43 ± 0.03	0.51 ± 0.03
KTZ	0.22 ± 0.01	0.20 ± 0.01	0.11 ± 0.03	0.19 ± 0.01	2.01 ± 0.11	0.20 ± 0.01
	0.43 ± 0.01	0.41 ± 0.01	0.22 ± 0.00	0.41 ± 0.03	3.63 ± 0.01	0.41 ± 0.03

## Supplementary Materials

Supplementary Materials Figure 1: the HPLC-UV chromatogram of methanolic extract from* Asparagus aethiopicus* leaf: 1, gallic acid; 2, chlorogenic acid; 3, vanillic acid; 4, caffeic acid; 5, robinin; 6, rutin; 7, apigenin. Figure 2: the HPLC-UV chromatogram of methanolic extract from* Senna alexandrina* fruit: 1, gallic acid; 2, neochlorogenic acid; 3, protocatechuic acid; 4, epigallocatechin; 5, gentisic acid; 6, vanillic acid; 7, caffeic acid; 8, syringic acid; 9, 6-hydroxy-4-methylcoumarin; 10, benzoic acid; 11, cynaroside; 12, isoquercetin; 13, psoralene; 14, quercetin; 15 luteolin; 16, kaempferol; 17, isorhamnetin; 18, rhamnetin.
