# Supplementary material for: Bioactivities of Traditional Medicinal Plants in Alexandria
Source: Evid Based Complement Alternat Med. 2018 Jan 31;2018:1463579. doi: 10.1155/2018/1463579 (PMC5831234; doi:10.1155/2018/1463579)
Supplement: Supplementary Materials — Figure 1: the HPLC-UV chromatogram of methanolic extract from Asparagus aethiopicus leaf: 1, gallic acid; 2, chlorogenic acid; 3, vanillic acid; 4, caffeic acid; 5, robinin; 6, rutin; 7, apigenin. Figure 2: the HPLC-UV chromatogram of methanolic extract from Senna alexandrina fruit: 1, gallic acid; 2, neochlorogenic acid; 3, protocatechuic acid; 4, epigallocatechin; 5, gentisic acid; 6, vanillic acid; 7, caffeic acid; 8, syringic acid; 9, 6-hydroxy-4-methylcoumarin; 10, benzoic acid; 11, cynaroside; 12, isoquercetin; 13, psoralene; 14, quercetin; 15 luteolin; 16, kaempferol; 17, isorhamnetin; 18, rhamnetin. [file 1463579.f1.pdf]

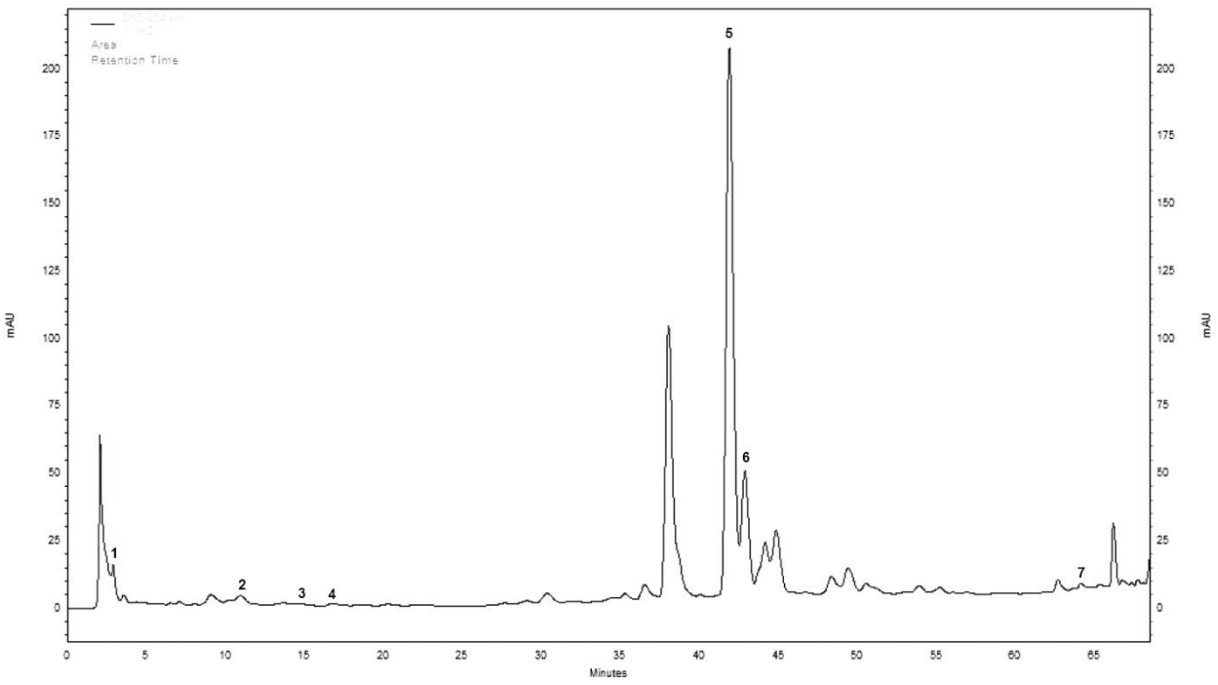

Supplementary Figure 1. The HPLC-UV chromatogram of methanolic extract from *Asparagus aethiopicus* leaf: 1 – gallic acid, 2 – chlorogenic acid, 3 – vanillic acid, 4 – caffeic acid, 5 – robinin, 6 – rutin, 7 – apigein.

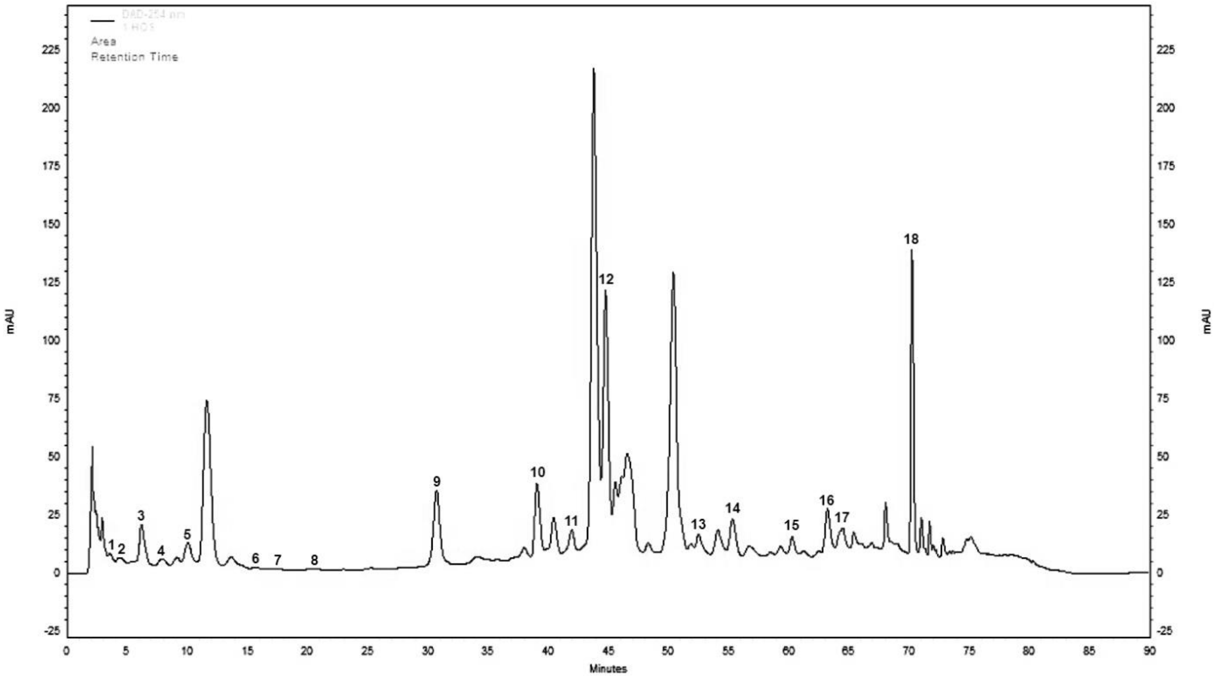

Supplementary Figure 2. The HPLC-UV chromatogram of methanolic extract from *Senna alexandriana* fruit: 1 – gallic acid, 2 – neochlorogenic acid, 3 – protocatechuic acid, 4 – epigallocatechin, 5 - gentisic acid, 6 - vanillic acid, 7 - caffeic acid, 8 - syringic acid, 9 - 6-hydroxy-4-methylcoumarin, 10 - bensoic acid, 11 – cynaroside, 12 – isoquercetin, 13 – psoralene, 14 – quercetin, 15 – luteolin, 16 – kaempferol, 17 – isoramnetin, 18 – rhamnetin.
